# Who represents me? A patient‐derived model of patient engagement via patient and family advisory councils (PFACs)

**DOI:** 10.1111/hex.12983

**Published:** 2019-10-23

**Authors:** Vadim Dukhanin, Scott Feeser, Scott A. Berkowitz, Matthew DeCamp

**Affiliations:** ^1^ Department of Health Policy and Management Center for Health Services & Outcomes Research Johns Hopkins Bloomberg School of Public Health Baltimore MD USA; ^2^ Johns Hopkins Medicine Alliance for Patients, LLC (JMAP) Baltimore MD USA; ^3^ Johns Hopkins Community Physicians, Remington Internal Medicine Baltimore MD USA; ^4^ Accountable Care Office of Johns Hopkins Physicians Baltimore MD USA; ^5^ Division of Cardiology, Johns Hopkins School of Medicine Baltimore MD USA; ^6^ Division of General Internal Medicine Johns Hopkins School of Medicine Baltimore MD USA; ^7^ Johns Hopkins Berman Institute of Bioethics Baltimore MD USA; ^8^ Center for Bioethics and Humanities and Division of General Internal Medicine University of Colorado Aurora CO USA

**Keywords:** health systems, participation, patient engagement, public involvement, representativeness

## Abstract

**Background:**

Despite increasing attention to patient and family advisory councils (PFACs), what patients who are not PFAC members expect of PFACs remains understudied. Understanding their expectations is critical if PFACs are to help health systems achieve certain outcomes (eg increased patient satisfaction with health systems).

**Objective:**

To obtain rich insights about what patients who are not PFAC members expect of PFACs.

**Design:**

From July to September 2018, we conducted a qualitative study using focus groups.

**Setting and participants:**

We recruited patients and caregivers who receive their care from the Johns Hopkins Medicine Alliance for Patients (JMAP), LLC, a Medicare accountable care organization that in 2014 established a PFAC, the Beneficiary Advisory Council.

**Approach:**

Using grounded theory, we analysed field notes, analytic memos and transcripts to develop a theoretical model of patient engagement via PFACs.

**Results:**

Forty‐two patients and caregivers participated in five focus groups that included individuals of different ages, races, health statuses and socio‐economic statuses. Participants were largely unaware of PFACs. Participants wanted to know *who* represented them (interpreted as a form of political representation) and emphasized the need for representatives’ diversity. *Who* mattered because *who* could affect *what* PFACs do. Participants expected that all patients should be able to communicate with PFACs and that meaningful engagement could enhance perceptions of health systems.

**Conclusions:**

Eliciting views about patient representation from patients who have not been engaged as advisors or representatives has the potential to inform PFACs’ activities. Attention should be given to improving and measuring patients’ awareness of, and interactions with, their patient representatives.

## 1 INTRODUCTION

Engaging patients in health‐care system design and quality improvement efforts is a critical part of patient‐centred care.^1^, ^2^, ^3^, ^4^ One way this is happening is via patient and family advisory councils (PFACs). PFACs are groups of patients, family members and family caregivers who serve as advisors in diverse health‐care settings, from individual clinics and hospitals to entire health systems. PFACs can be seen as part of a broader global movement over the past two decades towards patient and public involvement in health‐care planning.^5^, ^6^, ^7^

In the United States, PFACs are prevalent and may be increasing in number and diversity.^8^ This increase has been stimulated in part by formal regulatory requirements. For instance, some health‐care reforms, such as Medicare's Comprehensive Primary Care Plus (CPC+) or the new Maryland Primary Care Program (MDPCP), require practices to establish PFACs as a term of participation.

Toolkits exist to help facilitate the creation of PFACs.^9^, ^10^, ^11^, ^12^ However, PFACs’ potential may be underutilized. While case studies suggest how PFACs could improve the safety and quality of patient care, systematic and structured reviews have repeatedly demonstrated a lack of rigorous evaluation and evidence of such impact.^7^, ^13^, ^14^, ^15^ Much work remains to ensure PFACs can further patient‐ and family‐centred change in diverse health‐care settings.^16^, ^17^, ^18^, ^19^

Prior studies of PFACs and related participatory bodies have focused on the perspectives of PFAC members or members of the health‐care administrative teams with whom they liaise.^16^, ^17^, ^20^, ^21^ To our knowledge, few studies have assessed how a general patient population (ie non‐PFAC members) views PFACs. Engaging the broader patient population via PFACs may be an opportunity to improve patient‐centred care delivery, but there remains uncertainty about whether PFACs can or should play this role.^22^, ^23^, ^24^, ^25^ For instance, if PFACs are to facilitate achieving certain outcomes, such as those related to patient satisfaction with, or trust in, health‐care systems,^26^ PFACs may need to operate in ways that align with general patients’ expectations about these councils. To help fill this gap, we conducted a qualitative study investigating what patients who are not PFAC members might expect from a PFAC.

## 2 METHODS

### 2.1 Setting

This focus group study was conducted within the Johns Hopkins Medicine Alliance for Patients (JMAP), LLC, an accountable care organization (ACO) operating under the Centers for Medicare and Medicaid Services (CMS) Medicare Shared Savings Program since 2014. Medicare holds its ACOs accountable for the quality and cost of care delivered to a defined patient population. Serving patients in Maryland and the District of Columbia, JMAP has approximately 3000 physicians and health‐care providers who are responsible for 40 000 Medicare fee‐for‐service beneficiaries.

CMS requires its ACOs to include a patient representative, known as the ‘beneficiary representative’, on the ACOs’ governing boards. This must be someone who receives care from the ACO and who has no conflicts of interest. In collaboration with its first patient representative, JMAP additionally created a PFAC, known as the Beneficiary Advisory Council (BAC). The BAC is a volunteer group which is meant to reflect the diversity of JMAP's Medicare patient population. The BAC reviews policies, programmes and other initiatives, such as quality improvement efforts and patient outreach messages, with the goal of elevating the patient voice in JMAP decision making. This setting presents an opportunity to evaluate what patients who have not been engaged as representatives or advisors think of the concept of a PFAC in a real‐world scenario.

### 2.2 Sample

We recruited focus group participants from a sample of 429 JMAP patients who had indicated their willingness to provide deeper insights about their responses on a prior survey that assessed their awareness of patients representatives and how important patient representation is to them.^27^Of these 429, 169 received their care within JMAP's Greater Baltimore region. Focusing on these 169 was thought to improve the likelihood of participation, because focus groups were conducted in Baltimore City. However, because the Greater Baltimore region is defined by provider location (not patients’ addresses), our sample included participants from Baltimore City, Baltimore county (and neighbouring counties) and other outlying regions (eg individuals living in Pennsylvania who nevertheless receive care in Baltimore City). The sampling goal was to create diverse focus groups in terms of age, race, ethnicity, health and socio‐economic status. To do this, we collected demographic information during recruitment telephone calls and invited participants to join particular focus groups so that all groups would have variation across demographic characteristics.

### 2.3 Data collection

We held five focus groups between July and September 2018 at Johns Hopkins Berman Institute of Bioethics in Baltimore, Maryland. One member of the research team (MD) moderated, and another (VD) took detailed field notes. Each focus group lasted approximately 100 minutes and included 8‐9 participants. Focus groups were audio‐recorded and transcribed. For approximately two‐thirds of this time, we elicited participants’ views of patient advisory councils, the findings of which are reported here.

The research team created a focus group guide, informed by the research team's prior research,^4^, ^28^ to elicit participants’ expectations regarding PFACs. The guide underwent two rounds of substantive feedback from the BAC, resulting in a final focus group guide (see Appendix S1). Probing follow‐up questions (which could differ between groups, based upon each group's unique responses) elicited additional rich insights, and we modified the guide slightly over time to allow reflections and memos from earlier focus groups to inform later ones.

### 2.4 Data analysis

Data analysis utilized constant comparative techniques of grounded theory. Our approach drew upon Charmaz's constructivist version^29^ of Glaser and Strauss's classical grounded theory^30^ (without the procedural rigidity that many consider evident in Strauss and Corbin^31^). This approach recognizes that the researchers’ participation and own vantage points can affect the resulting theory and that the resulting theory is not abstract but tied to particularities of time and place. Analysis began with the field notes and memos created immediately after each focus group. Based on these notes and memos, it appeared that by the fifth focus group no significant new content was being elicited. Therefore, we placed recruitment on hold.

After the interviews were transcribed, open coding commenced. Two researchers (MD and VD) reviewed the transcripts and field notes, writing research memos and potential themes to explore during analysis. Next, one researcher (VD) reviewed the text line‐by‐line and developed a comprehensive set of codes, as close to the transcripts as possible, using both descriptive and evaluative coding approaches.^32^ We grouped related codes into preliminary categories based on emergent patterns in the data. A second researcher (MD) used this preliminary codebook to code transcripts independently, modifying some codes and reorganizing others. To help ensure intercoder reliability, the two researchers met and discussed code interpretation and code application discrepancies in detail, resolving them through discussion, and reviewed half of the transcripts line‐by‐line, for coding accuracy.

This process confirmed that no significant new content was being elicited by the fifth focus group. Having achieved reasonable thematic saturation, no additional sampling or recruiting occurred. Axial coding then commenced by re‐reviewing transcripts, field notes and memos using a constant comparative technique to clarify categories of codes, and to begin postulating relationships among them. Group‐to‐group validation, where a topic seemed important or of interest to all groups, was specifically sought. Specialized computer software was not needed. The full codebook, with codes, categories and example quotations, is available as Appendix S2.

The goal of our approach was to allow a core category and a theoretical model to emerge from the data. A core category is a central concept or main theme that applies to the research; it must appear frequently in the data, be abstract and help explain other categories and relationships.^31^ To arrive at a theoretical model, the researchers reviewed notes and memos, analysed the codes and categories from Appendix S2 in relation to the core category, and drew multiple diagrams of these relationships until arriving at a model that was most logically consistent with the data.^31^ To ensure reliability, the researchers employed reflexivity techniques and engaged in member checking by sharing both the full code book and the theoretical model with 20 focus group participants and with the members of the BAC. We used the received comments to update and finalize the codebook categories and the model.

### 2.5 Ethics review

This study was reviewed and approved by the Johns Hopkins Medicine Institutional Review Board on 26 March 2018 (IRB00167922).

## 3 RESULTS

### 3.1 Characteristics of participants

Of 169 potential people called in random order, 33 agreed to participate, 26 refused to participate, and 110 were not immediately reachable via the contact information left. In addition, we recruited seven family members and two friends of participants, which helped us meet our goal of including family caregivers. This recruitment yielded 42 total participants, comprised of both patients and caregivers who were not PFAC members.

Characteristics of the focus group participants are shown in Table 1. The focus groups were diverse in including individuals of various ages, races, levels of educational attainment, yearly personal income, self‐reported health statuses, self‐reported difficulty doing errands and statuses as caregivers or patients. Most participants were unaware of patient and family advisory councils in general and the BAC in particular. Additional details about participants, including each group's composition, are available as Appendix S3. Quotes below include individual's self‐identified gender and age.

**Table 1:** Demographics of patients who participated in focus groups (total N = 42)

Demographics	n (%)
Age	
35‐44	1 (2%)
45‐54	1 (2%)
55‐64	6 (15%)
65‐74	16 (38%)
75‐84	16 (38%)
84‐95	2 (5%)
Gender	
Male	19 (45%)
Race/ethnicity	
African American	11 (26%)
Caucasian	26 (62%)
Native American	1 (2%)
Other	4 (10%)
Hispanic/Latino	
No	42 (100%)
Educational attainment	
8th grade or less	1 (2%)
Some high school, but did not graduate	2 (5%)
High school graduate or GED	8 (19%)
Some college or 2‐year degree	4 (10%)
4‐year college graduate	11 (26%)
More than 4‐year college degree	16 (38%)
Yearly personal income	
<$25 000	13 (32%)
Between $25 000 and $50 000	9 (21%)
Between $50 000 and $100 000	10 (24%)
More than $100 000	9 (21%)
Not disclosed	1 (2%)
Overall self‐reported health	
Excellent	5 (12%)
Very good	11 (26%)
Good	17 (40%)
Fair	6 (15%)
Poor	2 (5%)
Not disclosed	1 (2%)
Patient reported being a caregiver	
Yes, currently	11 (26%)
Yes, in the past	3 (7%)
No	28 (67%)
Participant reported difficulty doing errands alone	
Yes	6 (15%)

### 3.2 Theoretical model

**Figure 1:**
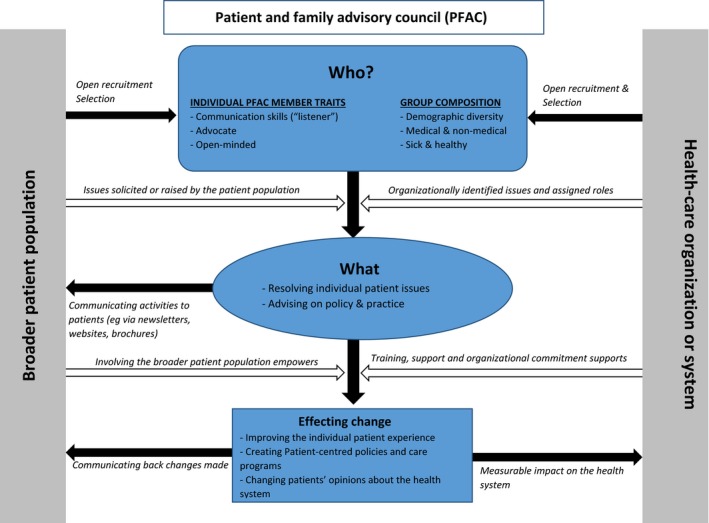
A conceptual model of patient and family advisory council (PFAC) engagement

The primary product of our analysis is the model shown in Figure 1. Because participants were largely unaware of the particular patient and family advisory council representing them, the model should be seen as an idealized conception of what patients might expect of a PFAC. This model places a PFAC at the interface between the broader patient population and the health‐care organization, emphasizing the clear expectation expressed by participants that a PFAC should be communicating bidirectionally with both. Below, we describe the core category and highlight relationships between other coded concepts.

### 3.3 Who: The core category

The core category of central, unifying importance among all focus groups was that of *who*. This was often the first response participants had when prompted to express their gut reactions to a patient advisory council. The word ‘who’ itself appeared on average more than once per page of the transcripts, and member checking with the BAC and several focus group participants confirmed the centrality of *who*. To illustrate:

I think who they are is very important. (female, 90)

That's true, yeah, who is representing me? (female, 36)

Related to the core topic of ‘who’ was a dominant expressed desire that patients who are not PFAC members should have a way to be *aware* of *who* is representing them on a council. Participants brainstormed ways of being informed, including mailed newsletters, websites and materials made available in medical practice offices, among others. As some participants said:

I would have to assume there would be willingness on the part of the advisory council is to have a pamphlet that talks about who these people are. (female, 76)

I think it's pretty bad that none of us knew anything about it. If we’re having somebody that's representing us and we didn't even know they’re around it seems like pretty poor, like [he] was saying, communication is lacking immensely. (female, 75)

It would interesting if more people knew about the council….(female, 71) (another participant agrees)…That’s the first thing… (female, 83) And then to see what would happen when people know about it. (female, 71)

Participants wanted to know who was on the council because they desired to *be able to connect with* them. Avenues for doing so included asynchronous forms of communication (such as online web forms, email, suggestion boxes at medical practice offices and telephone voicemail) and synchronous ones (such as face‐to‐face meetings with council members, town hall gatherings and allowing patients to sit in on council meetings).

So if I go back and Google… I'll be looking for, how would you contact them? (female, 70)

I should be able to contact a person on the council, the council shouldn't be something that's like it is now sort of off in the clouds… (female, 83)

One reason participants valued a PFAC interacting with the broader patient population appeared to be because they wanted to be sure the council was informed by others.

[As if addressing a council member.] Where do you get your information that you are bringing to the board? Who supplies that? Is it just your own personal important? Or are you talking to a lot of your friends? (male, 86)

This assumption about connecting with the council was so strong, that by the fifth focus group, the moderator challenged it. The moderator asked, ‘But [suppose] I'm one of the eight…and I'm a patient and I'm a volunteer…and we don't have time to be fielding complaints’. Various focus group participants responded vociferously, ‘Then you shouldn't be there!…Why be there?…Yeah, then why be there?..’

#### 3.3.1 Who: Who should be on a PFAC?

Focus group participants expressed views about who the ideal individual council member should be. Widespread agreement existed that PFAC members should have good communications skills (especially around listening, as different participants emphasized, being ‘a good listener’, ‘open‐minded’, or ‘understanding’, and having ‘compassion’). The ideal member should use those skills to advocate for others, with a ‘passion for helping’ and being ‘able to speak for other folks’. In addition, members should be open‐minded and not put forward only their own individual issues:

You definitely need somebody that could be objective because like one of you were saying about is it a problem? Or is it not a problem? (female, 75)

I think you have to go into it willing to look at the data available and make unbiased recommendations rather than this bad thing happened to me in the past that I need to get the system to correct it. (male, 71)

Participants also emphasized a certain amount of education and familiarity with the particular health‐care system whom the patient council advises. For instance, one participant said:

And I think they should also have familiarity with Johns Hopkins and the services that are provided now. And, in fact, really understand today's environment. (female, 70)

#### 3.3.2 Who: How should a PFAC be comprised?

When turning to the ideal PFAC composition, participants believed that individual PFAC members need not have every characteristic of an ideal member. Here, the emphasis was on diversity and balance. As participants said:

I don't think one patient probably could categorize everybody's illness, disability and all of that, I think we probably have to go a little deeper than one person. Because for instance, me I'm a paraplegic, and you have quads that can't move as much as I move, then you have bipolar… (male, 56)

Well if you go to try to help people you've got to get a wide diversity. (female, 90)

Diversity was defined by participants very broadly: it included not only a mix of age, gender, race, ethnicity, culture, socio‐economic status, overall health and disability status, but also experience and inexperience with health care (ie including PFAC members who might be retired health‐care professionals as well as PFAC members who might have more or less health‐care utilization). Several exemplary quotes are below:

It should be someone that has significant interaction with Johns Hopkins which usually means the disease of some degree of seriousness that requires them to come here frequently. (male, 70)

…and you might get different views of someone who's got 20 years of experience versus someone who just recently is suddenly introduced the hospital and medical care system and they might have very different perspectives…(female, 82) [another participant agrees]…Right, right, right, some people don't go for help… (female, 74)

For participants, diversity also involved ensuring PFAC members had different levels of familiarity and experience with PFACs; they recommended regular rotation of PFAC members and ‘term limits’ for members. Finally, participants emphasized how important it is, in an age of increasing electronic communication via email, patient portals and so on, to include patients with limited computer literacy; at the same time, participants recognized that technology could help facilitate geographic diversity through remote, web‐based access to PFAC meetings:

Well, there's not– once again, technology would offer the opportunity to be a board member to someone who is homebound if you use Skype or WebEx or some kind of meeting, offering diversity to those people as a representative on the council. (female, 70)

#### 3.3.3 Who: The broader patient population should help determine the ‘who’

The arrows in the model from the general patient population also reflect an expressed desire that the non‐PFAC members should help to determine who the PFAC is via the recruitment process. Some participants believed that the recruitment process should be open to anyone:

Perhaps there also could be, on the website there could be a listing of that board, of that volunteer board and information on it could be given, ‘This is basically what it is and what it does and if you're interested in serving with this group, call so and so.’ (female, 82)

However, most believed it would be necessary to screen potential PFAC members in some way, perhaps to ensure PFAC members had the characteristics previously discussed. Options discussed included selection by a physician who knows the patient, an interview process or even an election by the general patient population.

### 3.4 Why ‘who’ matters

As illustrated in Figure 1, the concept of *who* was important, in part, because *who* could affect *what* a council would do. The background of PFAC members could determine the types of issues that might come up or be considered important within the PFAC. One participant expressed this clearly:

So I think there's an interaction between who sits on the board and what kind of issues the board deals with, I think there's an interaction between the two and one affects the other. (female, 74)

Participants also recognized that a PFAC needs to be situated within the organizational structure in a way that it could make real decisions and have real impact. Reflecting on whether to join a PFAC, one participant said:

Personally, I would volunteer only if I had access top to bottom. To all of the different committees and how the decisions are made. I think myself to be more of the whole thing, not a part of it. (male, 82)

A participant from another group similarly noted:

I think to be effective it has to be part of the institutional structure. Otherwise, it just becomes a place where we vent our disagreements. (male, 78)

For participants, the roles and responsibilities that a PFAC takes on (whether because of its composition or how it is situated in the organization) were related to the kind of difference a PFAC could make (Figure 1, lower half). That was also influenced by the support given to a PFAC, including standing office space for meetings, staff support to aid with activities such as compiling input received from patients, creating newsletters and receiving calls. However, some participants cautioned that resources, though necessary, could compromise the perceived independence of a PFAC.

So I think what you were saying is that it doesn't feel like independent, if I say I'm getting everything from Johns Hopkins, pencil, papers, everything from Johns Hopkins, are we independent or will we start going toward…(female 1, 71)…[agreeing] Whose side are you on? (female 2, 71)…Exactly. Exactly. And even though we may be on the right side, how would the public see us? (female 1, 71)

Still, other participants thought that transparently disclosing the resources given to PFAC members could help mitigate the risk of being perceived as not independent:

Well you say that in the newsletter, ‘Hey, we need support to put our newsletter out,’ and transportation maybe, I don't know, whatever you need, right and you say …that doesn't make us not representing you, okay? (male, 80)

### 3.5 What: PFAC connecting back to patients to demonstrate and enhance impact

Besides making patients aware of a PFAC, reporting to patients on changes recommended and implemented by PFAC was important in two ways. First, to communicate PFAC’s effectiveness to the patient population broadly:

If you had a newsletter, let's say somebody called into a councilmember, they had a complaint, it ran its little course and the complaint was solved…then in a newsletter you could have the complaint and the solution …. (female, 83)

Well, I'd like to find out, is the meetings, is it public record, these quarterly meetings? So that I could see, yeah, this organization, this board does do something… (male, 80)

Second, connecting with the broader patient population was seen as a way to empower the PFAC:

Eight of them have eight perspectives and they don't know the 5 million‐5,000 others out there without listening to it…And I also think that the wider spread the exposure of the council, the more seriously the institution will take it. Because that's the way things work. If it's just eight people… (male, 78)

### 3.6 Effecting change: Effect of PFACs on views of the health system

Focus group participants were divided about whether a PFAC could affect their views of the health system. Some participants reacted positively to the idea of a patient council:

I think personally I would feel that it was being responsive to what I needed…I don't feel like I'm lost in this huge system because here is something that is representing me. (female, 75)

… I think is very positive and it's very important and certainly would enhance the standing of the university in my eyes or the hospital in my eyes. (female, 74)

It would make me feel as if they care … As if you had say … (female, 77)

However, it was more common across groups that positive perceptions of the health system depended upon awareness of who was on the PFAC, how they were chosen, what they were doing, what difference they were making and the ability to contact them:

Before it would change my opinion I would need to understand how they were appointed. How they’re functioning? Is it pro forma? Or is it a real group? (male, 74)

Yeah, what she was saying. They don't function. I mean they can have a board and everything but if they're not taking any input from them or trying to change anything what good are they? (male, 56)

I don't really know that much about what they do or what they could do in the future but if they did… (female, 74)

For others, the idea of a PFAC representing them was unlikely to change their view of the health system. Some were deeply sceptical of the whole idea; others were primarily concerned with their own patient‐physician relationships, and a PFAC did not seem capable of affecting them personally:

I'm kind of skeptical about the idea. It sounds great. But bureaucracies get entrenched in their own expertise and tend to discount the nonexperts who presumably the patient advisors would be. (male, 78)

If I'm taken care of the right way that's all I care, to tell the truth. (female, 90)

## 4 DISCUSSION

This study is, to our knowledge, one of the first to examine what patients and caregivers who are not members of patient and family advisory councils believe and expect regarding those councils. It has important implications for how PFACs are conceived and future research.

### 4.1 PFACs and political representation

The centrality of the concept of *who* to participants suggests their views could be interpreted through the theoretical concept of political representation. This was evident in the words used by participants (eg ‘term limits’ and, ‘Because when you have boards, or whatever you want to call them, representing people, that's just like the government. They're supposed to represent us’.)

At the same time, we cannot conclude that participants endorsed any particular view of representation. Pitkin's seminal work on political representation describes how representation aims to make the voices of the public ‘present again’ in policy processes.^33^ Pitkin describes four types of representation: formal representation (where an individual is authorized to be a representative, eg via election), descriptive representation (where the representative is supposed to have characteristics in common with those represented), symbolic representation (where the representative must be actually accepted as such by those represented) and substantive representation (where the representative is assessed by whether the interests of those represented are actually advanced).

Elements of descriptive representation (‘Does the PFAC member look like me/us?’) were evident in participants’ comments about diversity and whether PFACs represented all patients in the ACO. Indeed, evaluation based on descriptive representation has been used in the evaluation of PFACs.^17^ In addition, elements of formal representation appear evident based on comments related to how PFAC members are chosen (authorization) and how they communicate back to the patient population (accountability). Finally, participants’ interest in knowing what a PFAC does and what impact it makes suggest that they were also interested in substantive representation.

It is possible that participants quickly associate with representativeness because regulations sometimes use the term ‘patient representative’. We attempted to avoid that language by repeatedly using ‘advisory councils’. Still, our findings reinforced the importance of and need for additional clarity regarding representativeness. The need to understand legitimate representation is decades old, seen, for example, in health maintenance organizations in the 1970s^34^ and managed care organizations in the 1990s.^35^ One well‐studied context has been US Federally Qualified Health Centers (FQHCs) that provide care in underserved areas and have long required a majority of consumers on their governance boards.^17^, ^36^, ^37^, ^38^ Studies have shown that consumers on these boards are not representative of FQHC patients in the descriptive sense and, as a result, may fail to meet all patients’ needs equally; there is also evidence that increasing consumer representation may harm FQHC financial performance. Discussions about ‘who’ should be involved in health planning thus continue to be a major area of inquiry.^24^, ^39^

At a high level, however, our findings suggest participants endorsed a delegate model of representation (where representatives act on the stated preferences of those represented) as compared to a trustee model (where representatives follow their own thoughts regarding the right action).^40^ A delegate model requires PFACs to connect with their constituency, at least enough to learn their preferences.^16^, ^37^ In prior studies, PFAC members have themselves expressed the desire to learn more about the preferences of other patients.^19^

### 4.2 Power and control

We can also interpret our findings in relation to questions about how much power PFACs should have. Arnstein's ladder of participation describes eight levels of citizen participation over a policy process, ranging from manipulative non‐participation to consultation to full citizen control.^41^ As the model reflects, our participants appeared not to demand or expect full patient control. A minority expressed a concern that PFACs are tokenistic, using words such as ‘public relations’ or ‘window dressing’.

The reason for this could not be definitively inferred. Perhaps this resulted from perceived knowledge barriers; participants noted that having health‐care organization administrators involved with PFACs is essential for translating the complexities of modern health care to members. Alternatively, perhaps patients are conditioned not to expect such control. Our findings may cohere with recent scholarship suggesting that the top rung of the ladder, citizen control, may be an ideal that is difficult to be achieved.^42^

### 4.3 The impact of patient councils

Lastly, our findings intersect with recent efforts at improving evaluation of patient and public involvement in health care.^28^, ^43^ Although participants discussed elements of fair and inclusive decision‐making processes, as the model reflects, their primary interest was in PFACs’ ability to improve health‐care delivery. This included the patient experience of care delivery and concrete changes to policies and programmes.

Consistent with other work, our study suggests that PFACs could influence patient satisfaction and trust in the health system.^26^ Existing resources and toolkits emphasize the potential of engagement to improve trust,^10^ but real evidence is lacking.^44^ This is another critical knowledge gap, and it could be particularly important for vulnerable patients. In the United States (as elsewhere), distrust of the health‐care system may be associated with poor health as much as or even more than distrust of individual health‐care professionals,^45^ and evidence suggests that trust differs according to certain demographic variables, such as race.^46^

### 4.4 Implications

This study, by investigating the expectations of a general patient population (ie non‐PFAC members), adds important elements to the existing literature on patient engagement in health‐care governance. First, the components of the model can be the subjects of formal evaluation. In particular, this study underscores the important of metrics of communication between PFACs and the patients they represent. These have not been prominent in prior evaluation approaches.^28^, ^47^

Second, consistent with prior findings from a survey of more than 3000 Medicare beneficiaries,^27^participants believed that PFACs could improve perceptions of health‐care systems among their patients. Because patient perceptions and patient satisfaction matter for reputation, reflect care quality and can affect compensation under new payments models, PFACs may have untapped potential in this area. Importantly, our participants felt this could only happen if PFACs were *trustworthy*, based on who they are, what they do and what impact they have.

Third, our findings support several best practices recommended in existing patient engagement toolkits. This includes identifying and selecting patients for the PFAC in an open, transparent and structured manner; ensuring that a PFAC sets its own agenda and receives staff and resource support for its activities; engaging a PFAC across the organization (ie not being isolated from committees where ‘real’ decisions are made); and disseminating a structured report of PFAC performance back to patients broadly.

Other findings lend themselves to additional practical recommendations that health‐care system leaders and PFAC members can use to improve how PFACs operate. Increasing awareness of PFACs (which by some estimates is approximately 50%, among patients in general^27^) among non‐PFAC members may be the most important recommendation. Others include ensuring diversity in PFAC membership very broadly construed, rotating members over time, facilitating channels of communication between PFACs and the broader patient population, and evaluating PFAC effectiveness. These recommendations, which may be significant departures from how some PFACs typically operate in practice today, appear feasible for health systems to implement.

However, barriers to other expectations expressed by our participants must be acknowledged. Not all organizations may have the available time, personnel or financial resources to construct a PFAC using our proposed model. The idea of a general election of PFAC members is appealing but could be difficult to implement, given the lack of a defined or stable patient ‘electorate’ in many health systems. Similarly, although there existed a strong sense that patients in general should be able to view everything PFACs do, this could conflict with confidentiality agreements meant to permit free discussion at PFAC meetings and with the need to protect sensitive patient safety data.

### 4.5 Limitations

Like all studies, ours has limitations. First, although rich insights were obtained, the number of participants and focus groups was rather small and came from one specific geographic region. However, we were able to achieve thematic saturation and, likely because of participants’ lack of familiarity with any specific PFACs, also to obtain perspectives on an idealized version of PFACs. Future studies are needed to collect insights from patients who are aware of PFACs and to compare those study's findings to ours. Second, as a qualitative study, there is inherent subjectivity in analysis despite employed reflexivity and member checking; the findings may not generalize to all health‐care organizations or study populations. Third, by sampling and organizing participants into focus groups based on diversity, there is the possibility that findings might have changed if, for example, we conducted more homogenous groups. Fourth, although our groups included participants with diverse baseline views about patient representation via PFACs, there remains the possibility that participants’ views systematically differ compared to non‐participants.

We are careful not to conclude that this study's findings are the only correct theoretical model regarding what a PFAC should be or do. The fact that ‘who’ was the core category, rather than ‘why’, came somewhat as a surprise to the researchers (whose previous work had emphasized the ‘why’ question^4^). We acknowledge that this finding could have resulted from a circumstance where few patients know of PFACs’ existence. We must also acknowledge that some may view PFAC members not as representatives at all, but instead as lending their own individual experiences with care to organizational decisions. In fact, we observed this perspective while conducting semi‐structured interviews with ACO leaders and beneficiary representatives before the present study (unpublished data). Consistent with our constructivist approach to grounded theory, our findings represent a model created from the insights of a particular non‐PFAC member population.

## 5 CONCLUSION

This study represents one of the first in‐depth examinations of what a broader patient population expects of the patients who volunteer to serve as advisors at the health‐care system where they receive care. Patients who are not PFAC members expect that PFACs should expend significant efforts in communicating with all patients about who those councils are and what they do. By doing so, PFACs may be better situated to realize their potential to improve patient‐centred care and to improve general patients’ views of, and trust in, the health systems these councils advise.

## CONFLICT OF INTEREST

None.

## Data Availability

The data that support the findings of this study are available from the corresponding author upon reasonable request.

## References

[1] Berwick DM . What 'patient‐centered' should mean: confessions of an extremist. Health Aff (Millwood). 2009;28(4):w555‐565. 19454528 10.1377/hlthaff.28.4.w555

[2] Carman KL , Dardess P , Maurer M , et al. Patient and family engagement: a framework for understanding the elements and developing interventions and policies. Health Aff (Millwood). 2013;32(2):223‐231. 23381514 10.1377/hlthaff.2012.1133

[3] Coulter A . Patient engagement–what works? J Ambul Care Manage. 2012;35(2):80‐89. 22415281 10.1097/JAC.0b013e318249e0fd

[4] DeCamp M , Sugarman J , Berkowitz S . Meaningfully Engaging Patients in ACO Decision Making. Am J Accoutnable Care. 2015;3(2):30–33. PMC5647154 29057390

[5] Rowe G , Frewer LJ . A typology of public engagement mechanisms. Sci Technol Human Values. 2005;30(2):251‐290.

[6] Dumez V , Boivin A . A Canadian take on the international patient engagement revolution. Healthc Q. 2018;21(Special Issue):1‐6. 10.12927/hcq.2018.25643 30566399

[7] Sharma AE , Knox M , Mleczko VL , Olayiwola JN . The impact of patient advisors on healthcare outcomes: a systematic review. BMC Health Serv Res. 2017;17(1):693. 29058625 10.1186/s12913-017-2630-4 PMC5651621

[8] Sharma AE , Knox M , Peterson LE , Willard‐Grace R , Grumbach K , Potter MB . How is family medicine engaging patients at the practice‐level?: a national sample of family physicians. J Am Board Fam Med. 2018;31(5):733‐742. 30201669 10.3122/jabfm.2018.05.170418

[9] Institute for Patient and Family‐Centered Care . Reports and roadmaps. http://www.ipfcc.org/resources/downloads.html. Accessed December 6, 2018.

[10] Agency for Healthcare Research and Quality (AHRQ) . Strategy 1: Working with Patients and Families as Advisors. Rockville, MD: Agency for Healthcare Research and Quality; 2013.

[11] American Medical Association . Forming a Patient and Family Advisory Council. AMA STEPS Forward https://edhub.ama-assn.org/steps-forward/module/2702594. Accessed August 30, 2016.

[12] Abelson J , Li K , Wilson G , Shields K , Schneider C , Boesveld S . Supporting quality public and patient engagement in health system organizations: development and usability testing of the Public and Patient Engagement Evaluation Tool. Health Expect. 2016;19(4):817‐827. 26113295 10.1111/hex.12378 PMC5152717

[13] Cene CW , Johnson BH , Wells N , Baker B , Davis R , Turchi R . A narrative review of patient and family engagement: the "foundation" of the medical "home". Med Care. 2016;54(7):697‐705. 27111748 10.1097/MLR.0000000000000548 PMC4907812

[14] Liang L , Cako A , Urquhart R , et al. Patient engagement in hospital health service planning and improvement: a scoping review. BMJ Open. 2018;8(1):e018263. 10.1136/bmjopen-2017-018263 PMC5829665 29382676

[15] Oldfield BJ , Harrison MA , Genao I , et al. Patient, family, and community advisory councils in health care and research: a systematic review. J Gen Intern Med. 2019;34(7):1292‐1303. 30051331 10.1007/s11606-018-4565-9 PMC6614241

[16] Peikes D , O'Malley AS , Wilson C , et al. Early experiences engaging patients through patient and family advisory councils. J Ambul Care Manage. 2016;39(4):316‐324. 27576052 10.1097/JAC.0000000000000150

[17] Sharma AE , Huang B , Knox M , Willard‐Grace R , Potter MB . Patient engagement in community health center leadership: how does it happen? J Community Health. 2018;43(6):1069‐1074. 29777334 10.1007/s10900-018-0523-z

[18] Caplan W , Davis S , Kraft S , et al. Engaging patients at the front lines of primary care redesign: operational lessons for an effective program. Jt Comm J Qual Patient Saf. 2014;40(12):533‐540. 26111378 10.1016/s1553-7250(14)40069-2 PMC4484890

[19] Johnson KE , Mroz TM , Abraham M , et al. Promoting patient and family partnerships in ambulatory care improvement: a narrative review and focus group findings. Adv Ther. 2016;33(8):1417‐1439. 27352378 10.1007/s12325-016-0364-z PMC4969329

[20] Abelson J , Forest PG , Eyles J , Casebeer A , MacKean G , TEPCT . Will it make a difference if I show up and share? A citizens' perspective on improving public involvement processes for health system decision‐making. J Health Serv Res Policy. 2004;9(4):205‐212. 15526385 10.1258/1355819042250203

[21] Martin GP . Representativeness, legitimacy and power in public involvement in health‐service management. Soc Sci Med. 2008;67(11):1757‐1765. 18922611 10.1016/j.socscimed.2008.09.024

[22] Church J , Saunders D , Wanke M , Pong R , Spooner C , Dorgan M . Citizen participation in health decision‐making: past experience and future prospects. J Public Health Policy. 2002;23(1):12‐32. 12013713

[23] Li KK , Abelson J , Giacomini M , Contandriopoulos D . Conceptualizing the use of public involvement in health policy decision‐making. Soc Sci Med. 2015;138:14‐21. 26043073 10.1016/j.socscimed.2015.05.023

[24] Abelson J , Forest PG , Eyles J , Smith P , Martin E , Gauvin FP . Deliberations about deliberative methods: issues in the design and evaluation of public participation processes. Soc Sci Med. 2003;57(2):239‐251. 12765705 10.1016/s0277-9536(02)00343-x

[25] Thurston WE , MacKean G , Vollman A , et al. Public participation in regional health policy: a theoretical framework. Health Policy. 2005;73(3):237‐252. 16039343 10.1016/j.healthpol.2004.11.013

[26] Lee TH , McGlynn EA , Safran DG . A framework for increasing trust between patients and the organizations that care for them. JAMA. 2019;321:539‐540. 30676628 10.1001/jama.2018.19186

[27] DeCamp M , Dukhanin V , Hebert LC , Himmelrich S , Feeser S , Berkowitz SA . Patients' Views About Patient Engagement and Representation in Healthcare Governance. J Healthc Manag. 2019;64(5):332–346. 31498210 10.1097/JHM-D-18-00152 PMC6865800

[28] Dukhanin V , Topazian R , DeCamp M . Metrics and evaluation tools for patient engagement in healthcare organization systematic review. Int J Health Policy Manag. 2018;1‐15. 30316241 10.15171/ijhpm.2018.43 PMC6186472

[29] Charmaz K . Constructing Grounded Theory: A Practical Guide through Qualitative Analysis. London: Sage; 2006.

[30] Glaser BG , Strauss AL . The Discovery of Grounded Theory: Strategies for Qualitative Research. Chicago, IL: Aldine; 1976.

[31] Corbin J , Strauss A . Basics of Qualitative Research, 4th ed Thousand Oaks, CA: SAGE Publications, Inc.; 2015.

[32] Saldana J . The Coding Manual for Qualitative Researchers, 3rd ed Thousand Oaks, CA: SAGE Publications Ltd.; 2016.

[33] Pitkin H . The Concept of Representation. Berkeley, CA: University of California; 1967.

[34] Koseki LK . Consumer participation in health maintenance organizations. Health Soc Work. 1977;2(4):50‐69. 590868 10.1093/hsw/2.4.50

[35] Pearson SD , Sabin JE , Emanuel EJ . No Margin, No Mission. New York, NY: Oxford University Press; 2003.

[36] Wright B . Consumer governance may harm health center financial performance. J Prim Care Community Health. 2013;4(3):202‐208. 23799708 10.1177/2150131913475818 PMC5590748

[37] Wright B . Do patients have a voice? The social stratification of health center governing boards. Health Expect. 2015;18(3):430‐437. 23432950 10.1111/hex.12059 PMC5060786

[38] Wright B . Who governs federally qualified health centers? J Health Polit Policy Law. 2013;38(1):27‐55. 23052684 10.1215/03616878-1898794 PMC5602556

[39] Wiseman V , Mooney G , Berry G , Tang KC . Involving the general public in priority setting: experiences from Australia. Soc Sci Med. 2003;56(5):1001‐1012. 12593873 10.1016/s0277-9536(02)00091-6

[40] McCrone DJ , Kuklinski JH . The delegate theory of representation. Am J Pol Sci. 1979;23(2):278‐300.

[41] Arnstein SR . A ladder of citizen participation. J Am Plann Assoc. 1969;35(5):216‐224.

[42] Hoffman B , Tomes N , Grob R , Schlesinger M . Patients as Policy Actors. New Brunswick, NJ: Rutgers University Press; 2011.

[43] Boivin A , L'Esperance A , Gauvin FP , et al. Patient and public engagement in research and health system decision making: a systematic review of evaluation tools. Health Expect. 2018;21(6):1075‐1084. 30062858 10.1111/hex.12804 PMC6250878

[44] Ozawa S , Sripad P . How do you measure trust in the health system? A systematic review of the literature. Soc Sci Med. 2013;91:10‐14. 23849233 10.1016/j.socscimed.2013.05.005

[45] Armstrong K , Rose A , Peters N , Long JA , McMurphy S , Shea JA . Distrust of the health care system and self‐reported health in the United States. J Gen Intern Med. 2006;21(4):292‐297. 16686803 10.1111/j.1525-1497.2006.00396.x PMC1484714

[46] Boulware LE , Cooper LA , Ratner LE , LaVeist TA , Powe NR . Race and trust in the health care system. Public Health Rep. 2003;118(4):358‐365. 12815085 10.1016/S0033-3549(04)50262-5 PMC1497554

[47] Rifkin SB , Muller F , Bichmann W . Primary health‐care – on measuring participation. Soc Sci Med. 1988;26(9):931‐940. 3388072 10.1016/0277-9536(88)90413-3

