# Supplementary material for: Who represents me? A patient‐derived model of patient engagement via patient and family advisory councils (PFACs)
Source: Health Expect. 2019 Oct 23;23(1):148–58. doi: 10.1111/hex.12983 (PMC6978862; doi:10.1111/hex.12983)
Supplement: Supplementary file 1 [file HEX-23-148-s001.docx]

**Appendix 1. Focus Group Guide**

**Introduction**

The recording device is now on. Hello, my name is [PI/Other Co-Investigator/Trained Facilitator], and I will be facilitating our discussion today. Thank you for agreeing to participate.

We are here today because we want to learn about your personal experience with health care and to understand what you think about involving patients in the decisions hospitals and health systems make. You taking part because you filled out a survey about the Beneficiary Advisory Council (BAC), a group of patients that advises the [BLINDED ACO NAME]. In that survey, you indicated you would be willing to talk to us more.

Please feel free to interrupt me at any time if you have questions.

I want to reiterate a few key points:

First this discussion is confidential. So, you should not tell other people about what we discussed. You may choose to use a fake name when introducing yourself and on your name tent, if that would make you feel more comfortable.

Second, please remember that there are no right or wrong answers. Please be as candid as possible. This is not a debate, so you need not agree or challenge others. Let us hear your thoughts.

Third, you do not have to answer any question you do not want to. If you have thoughts that you’d rather share directly, or at a later time, you can also call [BLINDED NUMBER]. I will provide that number again later.

Do you have any questions before we get started?

**Participants’ Introductions**

Let’s begin by introducing ourselves. Please give your first name, or feel free to use a fake name.

*(Name tents will be provided. Ensure all participants introduce themselves.)*

**Explaining JMAP**

It may help to talk a bit about why this is important right now, to set the stage for our conversation.

You are here today because you are on Medicare and receive at least some of your medical care from [BLINDED ACO NAME AND AFFILIATES[. Your doctor is part of the [BLINDED ACO NAME]. [BLINDED ACO NAME] wants to bring YOU into the process of giving you the right care at the right time. I don’t want you to get caught up in all the details. [BLINDED ACO NAME] basically tries to support your doctors and other health care providers by coordinating care, designing new ways to provide care (for example, to better control blood pressure or encourage mammograms or colon cancer screening), and so on. For one reason or another, you may not be receiving these services, but we are still interested in your opinion. I also want to emphasize that I (the moderator) am not part of [BLINDED ACO NAME]. I will share what we discuss with [BLINDED ACO NAME] later, but I don’t be sharing what we discuss with [BLINDED ACO NAME] later.

*Below are the key interview domains we will cover. Because we cannot control or predict in advance what participants will say, other questions may be asked based on responses to these guiding questions.*

**Impressions of [BLINDED ACO NAME] Services**

INTRO PROMPT: I’ve just described [BLINDED ACO NAME] and its goals. You might remember that, on the original survey we asked you lots of questions. Some of these were about the services you might like to see [BLINDED ACO NAME] offer you. Today we want to learn more about that.

- **The Top 3 Services: Urgent Appointments, Care Coordination, and Online Portals.**
- Access to urgent appointments with specialists was one possible service.
  - What thoughts come to mind about urgent appointments?
  - For example, how soon is “urgent”? Or, what specialists should be included?
- Care coordination services help coordinate care across multiple doctors and care teams.
  - What do you feel about care coordination?
  - What are the ideal characteristics of a care coordinator?
- Communicating with your doctors or care teams though an online portal is also possible.
  - What is your first impression or gut reaction to having an online portal?
- **A Service Thought to be Less Valuable: Video Visits**
- Video visits are another possible service for patients. For a video visit, instead of going to the doctor’s office, you would communicate with your doctor or care team though a computer screen and microphone. .
  - What do you think or feel about this? (*probe deeply into barriers and facilitators for this; barriers might include privacy concerns or being unfamiliar with technology; facilitators could relate to being seen sooner, or in a more convenient location at home)*
- **A Service that Many Liked and Others Didn’t: Behavioral therapy**
- How would you feel about having additional services in the office to support you in managing stress, memory problems or depression? On the survey, we noticed that some patients would be very likely to use psychiatric therapy and counseling at their primary care provider’s office, but others would not.
  - What you do you think or feel about this? (*probe deeply into barriers and facilitators for this)*
- **Closing Question**
- If you had the opportunity to speak with leadership of [BLINDED ACO NAME] to offer suggestions for improvement, what would you say?

**Explaining the BAC**

We have just discussed [BLINDED ACO NAME]. Now I want to shift gears a bit.

Medicare requires all ACOs, like [BLINDED ACO NAME], to have a patient on the board that helps make decisions about how to provide health care better. [BLINDED ACO NAME] decided to have a “Beneficiary Advisory Council” or “BAC.” As [BLINDED ACO NAME]’s website says, the BAC is “a volunteer group of [BLINDED ACO NAME] patients that reflects the diversity of our Medicare patient population.” The BAC has eight members, and one member reports BAC suggestions back to the [BLINDED ACO NAME] board. The BAC assists in meeting [BLINDED ACO NAME]’s goals to improve the quality and value of care by reviewing policies, advising on educational materials, and contributing patients’ views at [BLINDED ACO NAME] meetings. [BLINDED ACO NAME] continues to work to identify ways to elevate the voice of patients, their families, and caregivers in the care process.

**Patient Participation at the Board Level**

INTRO PROMPT: Now that I’ve given you some background about this patient council, we want you to think broadly about what it might mean to you to have a patient council like this one.

- **First Impressions**
- Can you tell me what your first impression or “gut reaction” is to a patient council that provides input about your care? Describe how you think it could help improving the health care services we just discussed.
- Does having a Council affect what you think about Johns Hopkins or [BLINDED ACO NAME]?
- **The Ideal Patient Council Member**
- Describe to me, in your own words, an ideal patient council member.
  - What characteristics do you think would be important?
- How do you think patient council members should be chosen? *(If no ideas, provide examples: an application process, a vote, “friend of a friend,” etc.)*
- How many people should be on the Council?
- **What should a Council do?**
- What do you expect a patient council should be doing?
  - Council members are volunteers. What resources do you think a volunteer council needs?
- What one piece of advice would you have for developing an effective patient council?
- Think about another industry where consumer input is important. Can you describe how that input improves that industry? Is that like or not like patient input into a health care organization like [BLINDED ACO NAME]?
- **Interacting with a Council**
- In the survey you filled out, many people said they wanted to communicate with the patient council. How would you like to do that?
- If the Council were accepting new members, would you want to join? Why or why not?

**Other Issues**

Are there other issues related to these topics that we did not discuss today that you think are important?

Remember, if you have any other thoughts or comments to share later, you can do so by calling at 1-855-390-5803. Thank you for your participation. I will now turn off the recording device.
