# Supplementary material for: Who represents me? A patient‐derived model of patient engagement via patient and family advisory councils (PFACs)
Source: Health Expect. 2019 Oct 23;23(1):148–58. doi: 10.1111/hex.12983 (PMC6978862; doi:10.1111/hex.12983)
Supplement: Supplementary file 2 [file HEX-23-148-s002.docx]

| **Topic** | **Subtopic** | **Description** | **Illustrative quote or quotes** |
| --- | --- | --- | --- |
| Making a difference | Follow-up on the PFAC activities | Health care organization or system acts upon the PFAC recommendation | *“Do they feel as though any of what they’re saying is being heard? And if it's being heard is there action as a result of that?”* |
|  | Broader patient population involvement increases PFAC influence | PFAC influence can be increased if more patients are involved into the PFAC work | *“The wider spread the exposure of the council, the more seriously the institution will take it.”* |
|  | Presentation of anecdotal findings by the PFAC | The PFAC can motivate change by lending credibility to individual patient issues or cases | *“And you said the doctor said to you, you’re just bringing made anecdotal stories but it hasn’t been proven?”*  *“Yeah. Not science-based”*  *“Yeah. And [BLINDED INSTITUTION NAME] is a research science-based institution. So maybe it would be good if the patient council could have access to even some MD PhD graduate students who could help them come up with quantifiable questions to spread amongst a wide variety of patients so that we could start answering these questions in a more scientific, substantiated”* |
|  | How receptive the other board members and the institution is to PFAC | Influence of the PFAC depends upon how the PFAC is perceived by other stakeholders, including: (1) individuals who work with the PFAC; (2) the broader organization or its bureaucracy; (3) intermediary stakeholders (not just leadership level or patients, but nurses and other staff across the system) | *“But they forget the people in between, the nurse’s aides, the nurses, the people on the phones. They somehow get lost and nobody quite tells them what's going on and how to do it better.”* |
|  | Some issues are beyond the PFAC control | Certain areas of concern may not be able to be changed by the PFAC or even the health system or organization (e.g., a broader federal law). | *“Although I also think that a board can't do everything and all issues cannot be resolved and sometimes things that have to do with a patient in a hospital are affected by the city, by the state, by the federal government.”* |
|  | Access top-to-bottom across the organization | Ensuring the PFAC is integrated across all levels and committees of the organization | *“Unless there's a line of communication like the top of the hospital. If the president of [BLINDED INSTITUTION NAME] Medicine goes to the patient council and says, "If you don't get the reaction that you want come to me. Let me know. And I'll lay the law down.“* |
|  | Real or pro-forma? | Questioning if the PFAC is set to make a difference or only to formally fulfil the legislative requirement to have documented patient involvement | *“How they’re functioning?  Is it pro forma? Or is it a real group? Because I've seen too many organizational boards not to be functional groups. I mean they can have a board and everything but if they're not taking any input from them or trying to change anything what good are they?”* |
| PFAC recruitment and membership | Nominations by physicians | PFAC candidate members are nominated by a physician who knows them | *“A suggestion I might make that you might think about would be to have your primary care physicians nominate candidates because they know their patients well. They know who's been around for a while”* |
|  | Call for volunteers | PFAC candidate members are self-nominated | *“Perhaps there also could be, on the website there could be a listing of that board, of that volunteer board and information on it could be given, "This is basically what it is and what it does and if you're interested in serving with this group, call so and so."* |
|  | Election (not specified) | PFAC candidate members are nominated and selected via an unspecified election process | *“Maybe you should have an election. If they know about it you put 10 people up there and have them discuss their backgrounds and how they feel they should help patients and have a vote set on it. But that's not going to ever happen.”* |
|  | Selection via interviewing | Interviews are used to vet potential PFAC members for desired characteristics | *“Why do you think you're a candidate to be on the board?”*  *“Yeah, why would you want to serve on here, what do you think you could bring forth to this board?”* |
|  | Selection randomly | PFAC candidate members are chosen randomly from the pool after the nomination process | *“…how do you think they should be picked?*  *“Easy, by the computer.”* |
|  | Patient and their doctor are on a board simultaneously | Perceptions of the advantages and disadvantages of a PFAC member having his/her physician on the same board | *“It seems like you are getting the same-- you’re combining two votes in a sense. You’re diluting the diversity of the board because they are apt to agree with each other.”* |
|  | Membership is limited by terms | PFAC member have a set amount of time that they serve | *“I’d want the one year. I think, as we’re getting older, I wouldn't want a two or three year commitment.”* |
|  | Membership rotates | The PFAC involves a rotation of members over time | *“And having a rotation, too, is valuable. I know with most boards you’re elected to a board of directors for two years or three years.”* |
| Who | Who is my PFAC representative? | Discussing what is known about the concept of the PFAC | *“And I think patients should know who that patient advocate is …”*  *“So I think there's an interaction between who sits on the board and what kind of issues the board deals with, I think there's an interaction between the two and one affects the other”* |
| PFAC composition, including that the health care organization an PFAC should make efforts to facilitate inclusivity | Size | Determining the appropriate number of patient representatives or members of the PFAC, including number of PFACs by location or their function. | *“And there only eight? Maybe they need more. I would think they need more.”* |
|  | Representativeness and diversity reflected in the PFAC | Discussing that the composition of the PFAC should be representative of the patient population and/or reflect diversity of the patient population. (These were not always distinguished.) | *“I think if it's volunteer you’re going to get the diversity of the people that volunteered.”*  *“Well, they would be represented by of the people that volunteered to represent them.”* |
|  | Age | Discussing the age composition of the PFAC | *“Are there younger people on the beneficiary advisory council? Is that all younger people?”* |
|  | Gender | Discussing the gender composition of the PFAC | *“How many women? How many men?”* |
|  | Race or ethnicity | Discussing the racial composition of the PFAC | *“But I think it should be a mixture of races and ethnicities.”* |
|  | Cultural differences | Discussing how cultural differences will be reflected in the PFAC composition | *“But because of language barriers or mental barriers or whatever else financially maybe they couldn't afford to get here.”* |
|  | Socioeconomic status | Discussing how socioeconomic differences will be reflected in the PFAC composition | *“There is a different kind of patient council maybe that was staffed with patients who had run into financial difficulties and couldn't get the care that they needed.”* |
|  | Geographic diversity | For geographically dispersed systems, the PFAC should include members from different regions | *“Do you deal with graphic distribution in the council?”* |
|  | Health & disability status | Ensuring PFAC members have diverse health statuses | *“And what about hearing impaired”?* |
|  | Experience with health care | Ensuring PFAC members have a diverse set of prior interactions with the health system, including those who have none or few | *“It should be someone that has significant interaction with [BLINDED INSTITUTION NAME] which usually means the disease of some degree of seriousness that requires them to come here frequently.”* |
|  | Experience with sickness | Ensuring PFAC members have a history of experiencing sickness | *“I would ask the patient's that are selected to participate including the one sitting on the board be individuals who do you have some kind of extensive health problem.”* |
| Important personal traits for PFAC members | Ability to be objective or neutral; open-mindedness | Ensuring a PFAC member is able to be objective and is an open-minded person | *“I think you have to go into it willing to look at the data available and make unbiased recommendations rather than this bad thing happened to me in the past that I need to get the system to correct it.”* |
|  | Computer savviness | Discussing how being a computer savvy can be an obstacle to become a PFAC member or the degree it is necessary to fulfil the duties | *“So you might feel like it's excluding some people who don't use computers and that type of thing.”* |
|  | Education attainment level or intellectual capability | Discussing if a particular level of educational attainment or intellectual capability is needed to be a PFAC member | *“But somebody that can look at the situation that was presented to them and see how if it should be filtered into this group that Medicare wants to talk about, that they would be interested in. You don't need to have a college degree.”* |
|  | Medical background | Discussing if having a medical background precludes or helps to be a PFAC member | *“Also, remember that doctors are patients also so if somebody happens to be a doctor but is a patient too, I don’t see any reason why they couldn't be on a board…”* |
|  | Knowledge about the health care system or organization | Ensuring a PFAC member knows the system the PFAC operates in | *“And I think they should also have familiarity with [BLINDED INSTITUTION NAME] and the services that are provided now. And, in fact, really understand today's environment.”* |
|  | Good communication skills, particularly being a good listener | Ensuring a PFAC member has good communication skills | *“A good listener and a good talker.”*  *“Converse with doctors and administrators as equals.”* |
|  | What forms the PFAC member opinion / self-education | Discussing how a PFAC member is obtaining and absorbing the information they use for their PFAC duties | *“Is it just your own personal important? Or are you talking to a lot of your friends? How are you educated about your opinions that you want to offer to the board? Where is that coming from?”* |
|  | Being compassionate or sympathetic | Ensuring a PFAC member is a compassionate person | *“.. and you've got to be compassionate and understanding.”* |
|  | Ability to advocate for others | Ensuring a PFAC member is willing and capable to advocate for other patients | *“They definitely would have to be a person that goes around and asks other people.”*  *“They'd be able to speak for other folks, yeah ”* |
|  | Being a personally interested and reliable PFAC member | Ensuring a PFAC member is personally interested in their PFAC duties | *“To volunteer for something like that, you've got to be interested.”*  *““They should have a passion for helping…”* |
| PFAC roles and responsibilities | What? | General discussion of what is known about the PFAC functioning | *“What are they doing?”*  *“What issues to they deal with, yeah.”*  *“Tell us exactly what they're doing.”* |
|  | Acting upon input received from other patients | Discussions how acting upon the input from the general patient populations should be a part of the PFAC responsibilities | *“You call in a problem, right, to whoever and he is bound to take your complaint to the council table, okay, there they're going to hash it out whether it should be forwarded to the big guy to go between the thing or whatever, right, and that's what feedback, feedback is what it is.”* |
|  | Dealing with individual patient cases, both negative and positive | Discussions suggesting that the PFAC’s role is to work with individual patient experiences of the health care system or organization | *“And are they dealing strictly with complaints? Or with suggestions?”* |
|  | General organizational issues, not just individual complaints | Discussion suggesting that the PFAC role is to operate on the organizational level of policy and practice advising | *“We still haven't determined whether this is going to be an advocacy group or if they try to influence policy.”* |
|  | Not only identify problems, but also propose solutions | Discussions suggesting that PFAC is also involved in devising of the identified problems | *“Is this council supposed to find solutions? Or are they just supposed to say these are things that people are concerned about? And somebody else finds the solution?”* |
|  | Proactively find ways to improve care, not just react | Discussions suggesting that PFAC also employs proactive thinking | *“I would really like to see a group like that looking at things preventatively.”* |
|  | Advise on how funds should be used or distributed | Discussions on to what degree the PFAC should be involved in decisions regarding the health care system or organization’s finances | *“Yeah, the patient issue should be not financially related.”*  *“Because people can dream up all sorts of great recommendations that have no chance of happening because they financially are unreasonable.”* |
|  | Discussing the PFAC’s internal decision-making | Discussions on how the PFAC members come up with collective decisions | *“Like I said, just like jury duty, they have to come to one conclusion of anything.”* |
|  | Receive issues from the organization, not just identify on their own | Discussions on if the PFAC should receive items to consider from the health care system or organization | *“Does the ACO take issues to the advisory council? Or just the other way around?”* |
|  | Acknowledging PFAC members as volunteers and the amount of work required, including the frequency of meetings. | Discussions of the balance of PFAC members volunteering time and the expected workload | *“I think they would be overwhelmed when people realized …”*  *“It would depend upon how many issues are flying at one time.”*  *“That would be a lot of meetings for someone.”* |
|  | How fast is the PFAC available for patient input | Discussions on how fast the PFAC members should react to the emergent issues | *“Immediately, are you talking about immediately? He needed somebody immediately. Are they available?”* |
|  | Connection to Patient Relations office | Discussions about the distinction of the PFAC roles with the Patient Relations office(s) of the health care system or organization | *“Isn’t patient advocacy a group mainly for the inpatients. Because when I’ve been in the hospital they come around and talk to me.”* |
|  | Respecting confidentiality where necessary | Discussions on the issues of patient confidentiality that effect the PFAC functioning | *“Well I think there's a question of confidentiality, how you're talking to other patients and how that works with your relationship with your doctors.”* |
| PFAC connection with general patient population – | Meetings public records / open meetings | Ensuring that the PFAC meetings are either open to the public or have public records | *“Well, I'd like to find out is the meetings is it public record, these quarterly meetings, so that I could see, yeah, this organization, this board does do something.”* |
|  | Making the general patient population aware of the PFAC. | Via the pamphlets, website, newsletter, patient portal, physicians/physician offices, or radio/TV | *“It could be communicated when you get all of your HIPAA stuff and all of those things you have to sign when you go that says, "Here's your advocate." Or have something on MyChart to flag the advocate.”* |
|  | Getting input from the general patient population | Via the town halls, by having PFAC meetings open to the public or guests; one-on-one meetings with PFAC members; beneficiary surveys; focus groups with patients; hotlines or telephone; emails; weblogs/web forms; text; via the patient portal; or by employing a “secret shopper” | *“One of the ways that you could make the patient advisory council members accessible to-- I would have to assume there would be willingness on the part of the advisory council is to have a pamphlet that talks about who these people are . A mailing address or an email address where you … could contact the patient advisory council member so that they were getting feedback from the entire population”* |
|  | Communicating PFAC activities and performance back to the general patient population | Via the pamphlets, website, newsletter, patient portal, via physician/physician offices; town halls, by having PFAC meetings open to the public or guests; one-on-one meetings with PFAC members; automated calls; or emails | *“If you had a newsletter, let's say somebody called into a councilmember, they had a complaint, it ran its little course and the complaint was solved, the question was solved then in a newsletter you could have the complaint and the solution ….”* |
| Being a patient representative on Board | Perceptions of being the PFAC patient representative on the board | Perceptions of being the PFAC patient representative on the board of the health care system or organization | *“Probably intimidating. Depending on what your background is. If you’ve been in business or something you may be more comfortable going to a board but somebody else might be afraid to say anything.”* |
| Training of the PFAC members | Components of the training | Orientation including: mission statement; the PFACs role within the organization; the metrics to which the health system is held accountable; basics of healthcare financing; job description; | *“A very good orientation to work on the advisory council before I came on it..”*  *“Job description.”*  *“I think I would just like to see the org chart …”* |
| Providing resources to the PFAC while preserving independence | Resources need to sustain the PFAC | Resources including office space, support staff to aid with compiling input from patients, creating the newsletter, receiving calls, and other communications needs. | *“It could be a paid coordinator who is called by a patient and then they would refer it to this committee of eight.”*  *“… someone [who] could compile the views.”*  *“…a place or a room like an office, like a little office where we could meet each other… If you’re doing a job you have to have access to the environment you’re working in.”* |
| Benefits of the PFAC Success | Benefits to the organization or health care system | More channels from patients to the health system; mitigating negative health care events; ability to identify patterns of problems; learning about changes patients want and what patients need. | *“But I think it's a consensus of opinion that opening more channels of communication directly from the patients to the hospital would be a good thing.”*  *“I also think that you would be able to identify patterns. If the patient advisory council is receiving information that there's a pattern of problems just being able to enable the hospital to address the pattern would be successful.”* |
|  | Benefits to patients in general | In addition to organizational benefits, individual patients may benefit from feeling their voices matter (or that the system cares about them) or that there is a PFAC that will listen to them. | *“I don't feel like I'm lost in this huge system because here is something that is representing me.”*  *“… knowing that there is a beneficiary advisory council. And that they have access to that if they have issues.”* |
| Changes to patients’ views about the health care system or organization due to the PFAC | Yes | Having a PFAC changes their opinion positively (e.g., by making the organization feel smaller or because the system would seem more caring) | *“But in general, the concept of having patient input into the whole hospital process I think is very positive and it's very important and certainly would enhance the standing of the university in my eyes or the hospital in my eyes.”* |
|  | No | Having a PFAC would not change their opinion (e.g., because only individual care relationships matter) | *“If I'm taken care of the right way that's all I care to tell the truth.”*  *“… because [BLINDED INSTITUTION NAME] as a health system has become this huge octopus that's everywhere. So if you're taking this council you’re having one small part of [BLINDED INSTITUTION NAME], that's not going to change your whole perspective of a [BLINDED INSTITUTION NAME] system, I don’t think.”* |
|  | It depends | The change in their opinion would depend upon the PFAC’s function and performance | *“Before I change the opinion, I need to know more.”*  *“It depends on the council.”* |
