# Supplementary material for: Who represents me? A patient‐derived model of patient engagement via patient and family advisory councils (PFACs)
Source: Health Expect. 2019 Oct 23;23(1):148–58. doi: 10.1111/hex.12983 (PMC6978862; doi:10.1111/hex.12983)
Supplement: Supplementary file 3 [file HEX-23-148-s003.docx]

**Appendix 3 Demographics of the interviewed Medicare beneficiaries**

| Demographics | N = 42  n (%) | Group 1  N_1_ = 8 | Group 2  N_2_ = 8 | Group 3  N_3_ = 9 | Group 4  N_4_ = 8 | Group 5  N_5_ = 9 |
| --- | --- | --- | --- | --- | --- | --- |
| **Age** |  |  |  |  |  |  |
| 35-44 | 1 (2%) | - | - | - | - | 1 |
| 45-54 | 1 (2%) | 1 | - | - | - | - |
| 55-64 | 6 (15%) | 1 | 2 | 1 | - | 2 |
| 65-74 | 16 (38%) | 2 | 3 | 5 | 3 | 3 |
| 75-84 | 16 (38%) | 3 | 2 | 3 | 5 | 3 |
| 84-95 | 2 (5%) | 1 | 1 | - | - | - |
| **Gender** |  |  |  |  |  |  |
| Male | 19 (45%) | 3 | 4 | 5 | 4 | 3 |
| Female | 23 (55%) | 5 | 4 | 4 | 4 | 6 |
| **Race/ethnicity** |  |  |  |  |  |  |
| African American | 11 (26%) | 3 | 2 | 2 | 1 | 3 |
| Caucasian | 26 (62%) | 5 | 5 | 6 | 6 | 4 |
| Native American | 1 (2%) | - | - | - | - | 1 |
| Other | 4 (10%) | - | 1 | 1 | 1 | 1 |
| **Hispanic/Latino** |  |  |  |  |  |  |
| No | 42 (100%) | - | - | - | - | - |
| **Educational attainment** |  |  |  |  |  |  |
| 8th grade or less | 1 (2%) | 1 | - | - | - | - |
| Some high school, but did not graduate | 2 (5%) | 1 | 1 | - | - | - |
| High school graduate or GED | 8 (19%) | 1 | 1 | 2 | 1 | 3 |
| Some college or 2-year degree | 4 (10%) | - | 1 | 1 | 1 | 1 |
| 4-year college graduate | 11 (26%) | 4 | 1 | 1 | 1 | 4 |
| More than 4-year college degree | 16 (38%) | 1 | 4 | 5 | 5 | 1 |
| **Yearly personal income** |  |  |  |  |  |  |
| <$25,000 | 13 (32%) | 3 | 2 | 3 | 1 | 4 |
| Between $25,000 and $50,000 | 9 (21%) | 1 | 1 | 1 | 2 | 4 |
| Between $50,000 - $100,000 | 10 (24%) | 4 | 2 | 3 | 1 | - |
| More than $100,000 | 9 (21%) | - | 3 | 2 | 4 | - |
| Not disclosed | 1 (2%) | - | - | - | - | 1 |
| **Overall self-reported health** |  |  |  |  |  |  |
| Excellent | 5 (12%) | 1 | 1 | - | 1 | 2 |
| Very good | 11 (26%) | 2 | 1 | 3 | 3 | 2 |
| Good | 17 (40%) | 3 | 4 | 3 | 3 | 4 |
| Fair | 6 (15%) | 2 | - | 3 | - | 1 |
| Poor | 2 (5%) | - | 1 | - | 1 | - |
| Not disclosed | 1 (2%) | - | 1 | - | - | - |
| Beneficiary reported having  **Heart disease, high blood pressure, or stroke** |  |  |  |  |  |  |
| Yes | 22 (53%) | 7 | 2 | 6 | 3 | 4 |
| No | 19 (45%) | 1 | 6 | 2 | 5 | 5 |
| Not disclosed | 1 (2%) | - | - | 1 | - | - |
| Beneficiary reported having  **Diabetes** |  |  |  |  |  |  |
| Yes | 8 (19%) | 1 | 1 | 3 | 2 | 1 |
| No | 33 (79%) | 7 | 7 | 5 | 6 | 8 |
| Not disclosed | 1 (2%) | - | - | 1 | - | - |
| Beneficiary reported having (including history of)  **Cancer** |  |  |  |  |  |  |
| Yes | 9 (21%) | 4 | 1 | 1 | 2 | 1 |
| No | 32 (77%) | 4 | 7 | 7 | 6 | 8 |
| Not disclosed | 1 (2%) | - | - | 1 | - | - |
| Beneficiary reported having  **Depression or mental illness** |  |  |  |  |  |  |
| Yes | 7 (17%) | 2 | - | 1 | 1 | 3 |
| No | 34 (81%) | 6 | 8 | 7 | 7 | 6 |
| Not disclosed | 1 (2%) | - | - | 1 | - | - |
| Beneficiary reported being a  **Caregiver** |  |  |  |  |  |  |
| Yes, currently | 11 (26%) | 3 | 2 | 2 | 1 | 3 |
| Yes, in the past | 3 (7%) | - | - | 2 | - | 1 |
| No | 28 (67%) | 5 | 6 | 5 | 7 | 5 |
| Beneficiary reported  **Difficulty doing errands** alone |  |  |  |  |  |  |
| Yes | 6 (15%) | 1 | 1 | 2 | 2 | - |
| No | 36 (85%) | 7 | 7 | 7 | 6 | 9 |
